# Preparation of PEO/Clay Nanocomposites Using Organoclay Produced via Micellar Adsorption of CTAB

**DOI:** 10.1100/2012/270452

**Published:** 2012-12-10

**Authors:** Ahmet Gürses, Mehtap Ejder-Korucu, Çetin Doğar

**Affiliations:** ^1^Department of Chemistry Education, Kazım Karabekir Education Faculty, Atatürk University, 25240 Erzurum, Turkey; ^2^Department of Chemistry, Faculty of Science and Literature, Kafkas University, 36100 Kars, Turkey; ^3^Department of Science Education, Faculty of Education, Erzincan University, 24030 Erzincan, Turkey

## Abstract

The aim of this study was the preparation of polyethylene oxide (PEO)/clay nanocomposites using organoclay produced via micellar adsorption of cethyltrimethyl ammonium bromide (CTAB) and their characterisation by X-ray diffraction (XRD), and Fourier transform infrared (FT-IR) spectra, and the investigation of certain mechanical properties of the composites. The results show that the basal distance between the layers increased with the increasing CTAB/clay ratio as parallel with the zeta potential values of particles. By considering the aggregation number of CTAB micelles and interlayer distances of organo-clay, it could be suggested that the predominant micelle geometry at lower CTAB/clay ratios is an ellipsoidal oblate, whereas, at higher CTAB/clay ratios, sphere-ellipsoid transition occurs. The increasing tendency of the exfoliation degree with an increase in clay content may be attributed to easier diffusion of PEO chains to interlayer regions. FT-IR spectra show that the intensity of Si-O stretching vibrations of the organoclays (1050 cm^−1^) increased, especially in the ratios of 1.0 g/g clay and 1.5 g/g clay with the increasing CTAB content. It was observed that the mechanical properties of the composites are dependent on both the CTAB/clay ratios and clay content of the composites.

## 1. Introduction

Polymer/clay nanocomposites bring many superior advances in material engineering due to the low filler loadings of clay. These nano composites have been receiving an increasing amount of attention in several areas, such as manufacturing and universities [1–3]. Compared to raw polymer, using polymer/clay nanocomposites provides certain advantages such as mechanical properties (strength and elasticity modulus), optical properties (almost transparent), electrical properties (solid electrochemical battery), decreased gas and water permeability, and decreased flammability [4–6]. There are three different kinds of composite materials in existence; these are, respectively, polymer/clay composite, the intercalated polymer/clay composite, and the exfoliated or delaminated polymer/clay composite [7–10]. To obtain these composites, different methods are used in the literature. The first method is solution intercalation, the second is in situ intercalative polymerisation, and the last method is melt intercalation [4, 9]. Of all the methods, the solution intercalation method, which facilitates the production of not only thin films with polymer but also oriented-clay intercalated layers, was chosen for this study [11]. Polyethylene oxide (PEO) diffuses into the surface of the organo-clay modified with quaternary alkyl ammonium salts during intercalation. The quaternary alkyl ammonium salts are used to improve the wetting characteristics, to lower the surface energy of the inorganic component, and to be compatible with the polymer matrix [3, 12–15]. 

The aim of the study is to prepare PEO/clay nanocomposites using organo-clay produced via micellar adsorption of cethyltrimethyl ammonium bromide (CTAB). Prepared materials were characterised by X-ray diffraction (XRD), FT-IR spectra and then investigated for certain mechanical properties.

## 2. Materials and Methods

### 2.1. Materials Used

The materials used in this study were montmorillonite (MMT), polyethylene oxide (PEO), Cetyltrimethyl ammonium bromide (CTAB), and dichloromethane (methylene chloride). The montmorillonite, which is a commercial sample of bentonite from Çankırı deposit in Turkey was obtained from Karakaya Mineral Co., Ankara, Turkey. The cation exchange capacity (CEC) of the clayey soil sample was determined by the ammonium acetate method [16] (CEC: 107 meq/100 g, d_001_: 1.2620 nm). Chemical composition of the MMT was determined by X-ray fluorescence spectrometry. The results are given in Table 1. The polyethylene oxide (PEO) with a molecular weight of 900,000 g/mol was purchased from Aldrich. The cetyltrimethyl ammonium bromide (CTAB) and dichloromethane (methylene chloride) were purchased from Merck, and they were used without further purification. 

### 2.2. Preparation of Nanocomposites

#### 2.2.1. Preparation of Organoclay Samples

In order to expand the basal distance between clay layers and to produce organophilic clay, it is necessary to control interactions between the polymer matrix and clay particles. For this purpose, the montmorillonite (MMT) clay sample was modified with micellar adsorption of cethyltrimethyl ammonium bromide (CTAB) at the concentrations above critical micelle concentration (CMC) (0.1, 0.5, 0.7, 1.0, and 1.5 g/g clay). 

After a sample of 50.00 g MMT was dispersed in a 100 L shaker containing distilled water, the mixture was shaken in order to swell it at 200–250 rpm for 10 hours, and then CTAB above critical micelle concentrations were added to the clay suspension for 1 hour. The mixture was filtered, washed, and dried at 90°C under a vacuum and milled. 

#### 2.2.2. Preparation of Polyethylene Oxide/Organ Organo-Clay Nanocomposites

Polyethylene oxide/organo-clay nano composites were produced using the solution interaction method. The organically modified clay was dispersed and swollen in methylene chloride for 24hours in a three-necked flask at an ambient temperature and sonicated for 15 minutes. The PEO/methylene chloride ratio, 1 : 33.6 (g/mL), was maintained by adding an additional quantity of solvent as well as PEO. The flask and its contents, which were fitted with a condenser, a mechanical stirrer, and a thermometer pocket, were then placed in an oil bath heated to 75°C. The flask was kept under a stirring condition for 10hours and then the nano composites were put into Petri dishes and air dried overnight at an ambient temperature. The same procedure was applied to different clay ratios (2.0%, 4.0%, 5.0%, and 8.0%).

### 2.3. Characterisation of Nanocomposites

#### 2.3.1. X-Ray Diffraction (XRD)

The nanocomposites are analyzed by using a Rigaku D/MAX 2200/PC X-ray diffractometer instrument that employed K-*α* radiation (*λ* = 1.5404 Å) and performed at 2 to 40°. The scanning rate of the instrument was 2°/minute.

#### 2.3.2. Fourier Transform Infrared (FT-IR) Spectroscopy

FT-IR spectra of organo-clay and nano composites were taken on a Perkin-Elmer Spectrum-One, by KBr pelletization method from 4000 to 400 cm^−1^. 

#### 2.3.3. Tensile Testing

The mechanical properties of PEO/organo-clay nano composites, which include tensile strength, elasticity module, and strain percentage, were determined. For this reason, the PEO/organo-clay nanocomposites were die cut with a rectangular die and tested with a Shimadzu AG-100kNIS test instrument under ASTM 638 M-91a. All tests were carried out at 5 kN at an ambient temperature with a speed of 2 mm/minute. 

#### 2.3.4. Zeta Potential Measurements

The zeta potential values of the raw clay and organo-clay samples, prepared at different CTAB/clay ratios (0.1, 0.5, 0.7, 1.0 and 1.5), were measured using a Zeta Meter 3.0+. 

## 3. Results and Discussion

### 3.1. Zeta Potential and Interlayer Distance

**Figure 1:**
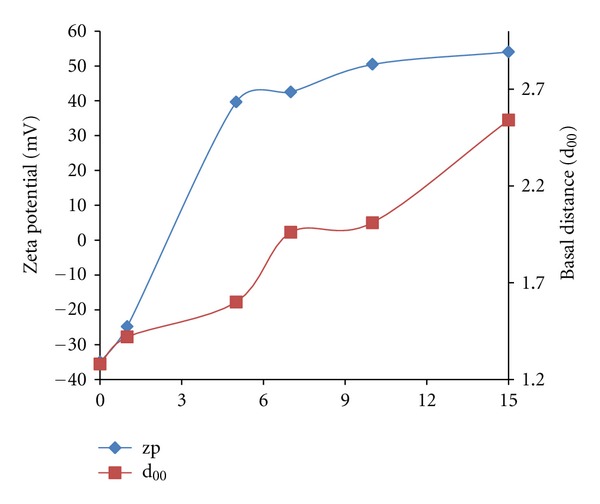
The change of interlayer distance and zeta potential values at various CTAB/clay ratios.

It is shown that the basal distance between the layers increased with the increasing CTAB/clay ratio as parallel with the zeta potential values of particles (see Figure 1). In this process, involving micellar adsorption, it is clear that not only the distances between the layers but also the change of zeta potential values are associated with the geometry of the micelle. Consequently, the aggregation number of micelles was calculated. 

This figure reveals that the interlayer distance after micellar adsorption of CTAB, in the case of 0.1 g CTAB/g clay ratio, was calculated as 1.42 nm from an XRD diffractogram. Taking this into consideration, the average radius of a spherical CTAB micelle is 1.3 nm and the interlayer distance in the raw clay is approximately 1.28 nm, indicating that we can infer that the formed micelles are spherical. 

As shown in Figure 1, the zeta potential values of particles increase with the increase in CTAB/clay ratio up to positive values. Since Tanford (1972) reported that the aggregation number of a spherical CTAB micelle was 215 and observations in the previous experiments show that the neutralisation of the surface charge of clay particles was provided in a 0.24 g CTAB/g clay ratio, showing that a CTAB/clay ratio of 0.1 g/g is not sufficient for neutralisation [17]. 

The change in the values of the particles' zeta potential with a 0.5 g CTAB/g clay ratio is approximately 2.1-fold compared to the raw clay. Accordingly, it can be said that micellar geometry is converted from spherical form to ellipsoidal oblate (*a*_0_/*b*_0_ = 1.25) with increasing the CTAB amount and the amount of aggregation was almost certainly 177.0. Similarly, it has been determined that the zeta potential values in the case of a 0.7 g CTAB/g clay ratio reach positive values and its change in quantity is approximately 2.2-fold compared to the raw clay. In this CTAB-clay ratio, the interlayer distance has extended to 1.96 nm (see Figure 1), indicating that the micelle geometry has converted to an ellipsoidal oblate form. If the form of lateral bilayer micelles adsorption had occurred, the interlayer distance would be expected to be approximately 3.9 nm. In addition, a 3-fold increase in zeta potential values shows that predominant micelle geometry is oblate form (*a*_0_/*b*_0_ = 1.50). 

In a 1.0 g CTAB/g clay ratio, the change in zeta potential values and interlayer distance are increased 2.24-fold and 1.55-fold, respectively. Accordingly, micelle geometry is an oblate form (*a*_0_/*b*_0_ = 1.75) and the amount of aggregation is 346.5. In the case of a 1.5 g CTAB/g clay ratio, the interlayer distance changed approximately twice and the zeta potential values of particles have also changed 2.54 times. These figures are extremely high compared to those of the 0.1 g CTAB/g clay ratio, in which spherical micellar adsorption occurs [18] and micellar geometry is an oblate form (*a*_0_/*b*_0_ = 2). 

As a result of, associated theoretical data and zeta potential values, the amount of aggregation of 254.5 corresponding to the oblate form of micellar CTAB (*a*_0_/*b*_0_ = 1.5) is approximately 2.5 times its spherical form and also the zeta potential value, corresponding to the adsorption of micelles, in the form of oblate micelles is found to be approximately 2.5-fold according to the zeta potential value measure of the spherical micellar adsorption. 

Accordingly, it can be said that measurements of zeta potential can be regarded as an indicator of sphere-ellipsoid transition:

(1) $$\begin{array}{c} \Delta \xi =15.7\Delta d+68.6\;\;R^{2}=0.960, \end{array}$$

where Δ*ξ* and Δ*d* are the difference between zeta potential values and changes in the interlayer distance of organo-clay as normalised, respectively. 

### 3.2. X-Ray Diffraction (XRD)

**Figure 2:**
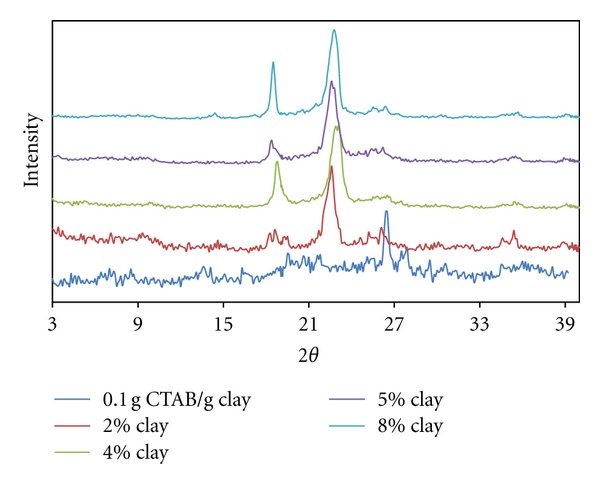
XRD patterns for produced PEO/clay nanocomposites at various clay compositions (0.1 g CTAB/g clay ratio).

Figure 2 depicts the fact that because of the solution intercalation of 98% PEO polymer with the organo-clay from spherical micellar adsorption with a g CTAB/g clay ratio, it can be argued that polymer chains intercalated into interlayer regions of clay and both tactoidal dispersions and the exfoliated dispersions abundantly occurred. 

Similarly, it is observed that the exfoliation degree increased with the increased clay content. It can be concluded that the increase in the exfoliation degree occurred because the increased clay content facilitates diffusion of PEO chains to interlayer regions. In addition, highly positively charged spherical micelles adsorbed into the interlayer region conceivably lead to deformation of the spherical micellar form after intercalating of helixical PEO chains. That is, it forces them into prolate form, and this deformation makes exfoliation easier. 

PEO diffusion can be thought of as an alternative mechanism that can force spherical micelles into lateral bilayer adsorption, which can cause exfoliation. It is possible that this alternative mechanism compensates for the increased clay content's negative effect in the degree of exfoliation. 

**Figure 3:**
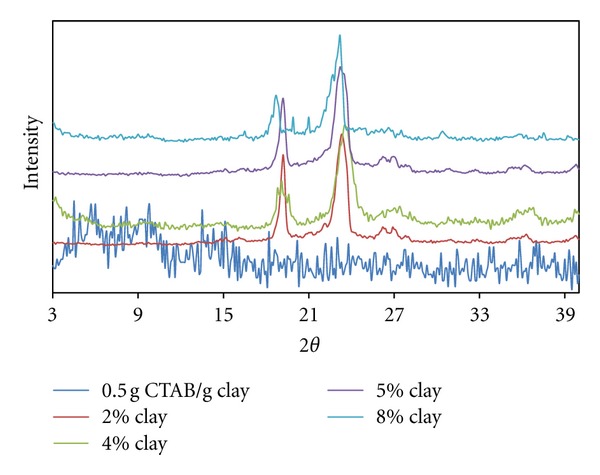
XRD patterns for produced PEO/clay nanocomposites at various clay compositions (0.5 g CTAB/g clay ratio).

In Figure 3 it can be seen that the PEO/clay composites with organo-clay in the case of 0.5 g CTAB/g clay contain exfoliated dispersions. Increased clay content facilitated the diffusion of PEO chains into the interlayer region; however, tactoidal dispersion partly occurs in 4.0% and 8.0% clay contents.

In this case, it can be thought that the diffusion of polymer chains with the increased clay initially deformed the micellar geometry to some extent, although later this effect was lessened in accordance with the increase in the number of clay particles. As the clay content increased, the probability of the emergence of the polymer chains' ellipsoidal transitions decreased in accordance with the increased particle numbers. 

**Figure 4:**
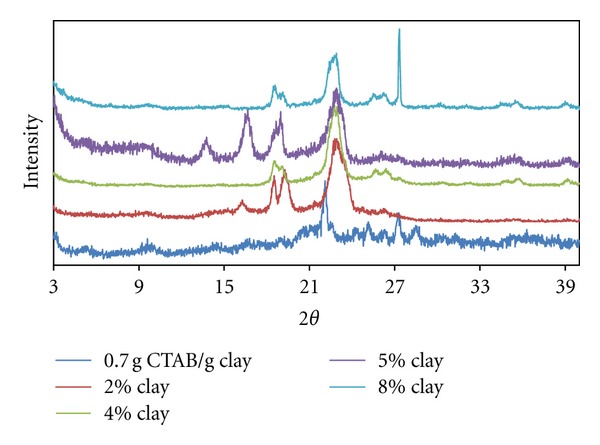
XRD patterns for produced PEO/clay nanocomposites at various clay compositions (0.7 g CTAB/g clay ratio).

In Figure 4, it can be clearly seen that the exfoliation degree with the increased clay amount increases in the produced organo-clay composites through micellar adsorption with ellipsoidal oblate in 0.7 g CTAB/g clay. However, it is noticed in the same figure that crystal peaks, possibly of polymeric origin, appeared in the clay content of 2.0% and 5.0%. 

In parallel to the decrease of the exfoliation degree in clay content of 5.0%, polymeric crystal peaks appeared and the interlayer distance relatively decreased. Accordingly, it can be argued that the diffusion of polymer chains in this clay content caused the ellipsoidal CTAB micelles to exchange with polymer chains. The micellar structures that distanced themselves from interlayer regions deformed the helixical structure of the chains in the polymer matrix, and thus the polymer chains led themselves to form different structures with different orientations. 

Conversely, in higher clay contents, the increase in the number of the interlayer regions where polymer chains can be diffused limited the polymer matrix outside the interlayer region. This significant increase in the exfoliation degree can be explained by the same polymer diffusion having forced adsorbed micelles into lateral bilayer structures. 

**Figure 5:**
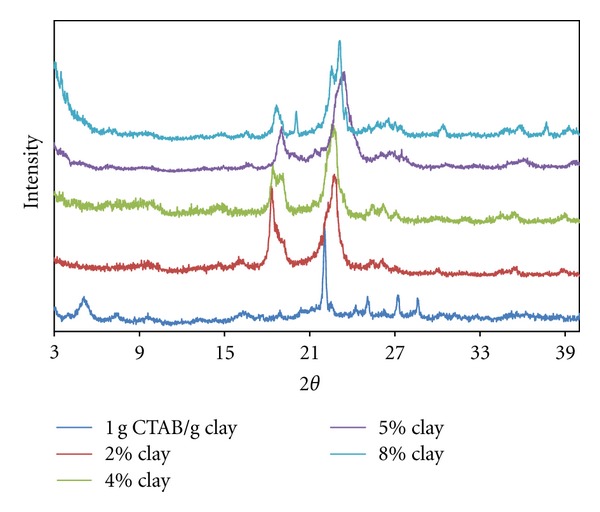
XRD patterns for produced PEO/clay nanocomposites at various clay compositions (1.0 g CTAB/g clay ratio).

In the samples of PEO/clay nano composites from organo-clay with 1.0 g CTAB/g clay, it is realised that both exfoliated and tactoidal dispersions emerged and that the degree of tactoidal dispersion increased, especially in higher clay contents (see Figure 5).

**Figure 6:**
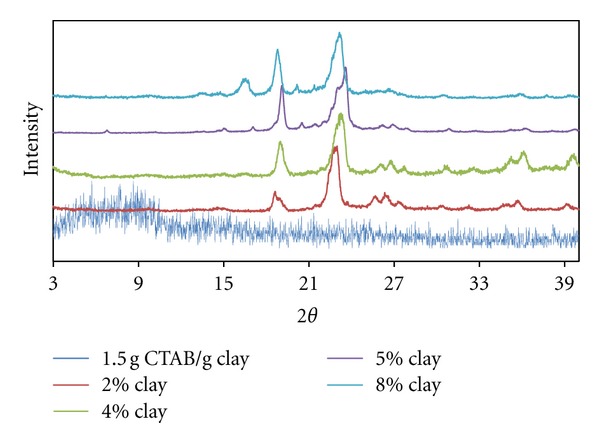
XRD patterns for produced PEO/clay nanocomposites at various clay compositions (1.5 g CTAB/g clay ratio).

In Figure 6 it can be clearly seen that in the PEO/clay nano composites samples with a 1.5 g CTAB/g clay ratio exfoliated structures emerged dominantly in almost all of the clay contents and that the interactions between the clay layers and polymer chains was highly effective. Higher aggregation numbers in high ratios of CTAB clay and amounts of oblated-micellar structures mean a higher increase in the diffusion of PEO chains into the interlayer region. 

### 3.3. FT-IR Analysis

**Figure 7:**
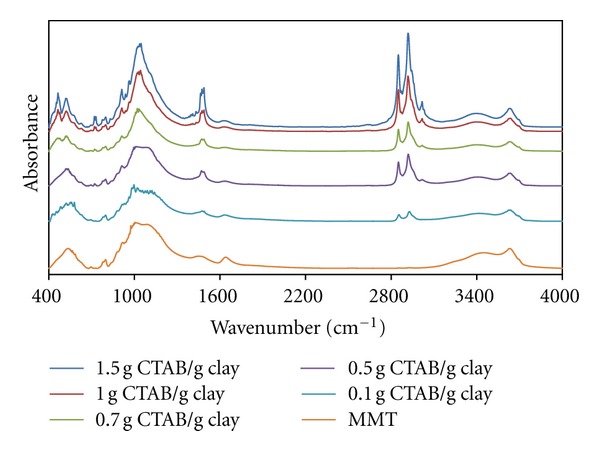
FTIR spectra of produced organoclays at various CTAB-clay ratios.

Figure 7 shows intensity of the infrared band at 1050 cm^−1^ associated with Si-O vibrations.

This increase can be correlated with the partial decrease of micelle-Si-O interaction, because of the increase in the aggregation number of micelles formed with the increasing CTAB content and their geometrical change. 

It can also be seen that the band observed at 1522 cm^−1^ associated with CH_2_ vibration, which slipped to the high-energy region, and the peak intensity increased with the increasing clay content. This can be explained by the transitions of spherical-ellipsoidal (oblate) micelles because of the increasing CTAB-clay ratio. 

It can be argued that small peaks corresponding to the swinging vibrations of CH_2_ groups emerged in ratios of 0.5 g CTAB/g clay and more. In accordance with the change in micellar geometry with the increasing CTAB-clay ratio, swinging vibrations of CH_2_ groups slipped left and the increase in the *a*_0_/*b*_0_ proportions of the micelles in oblate form caused a decrease in the energies of swinging vibrations. 

**Figure 8:**
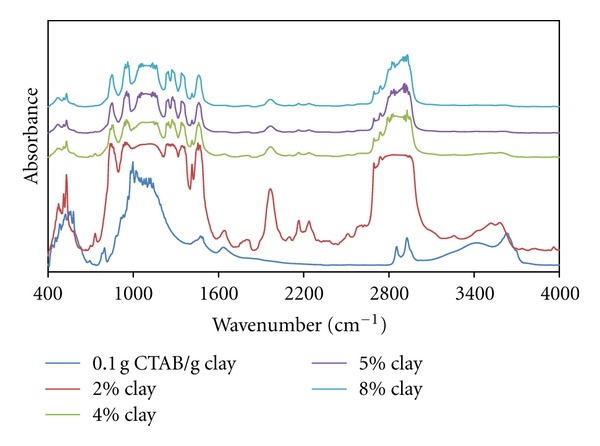
FTIR spectra of produced PEO/clay nanocomposites at various clay compositions (0.1 g CTAB/g clay ratio).

As can be seen in Figure 8, both bands appearing at 1800 cm^−1^ and 2400 cm^−1^ may be associated with CH_2_ vibration and scissoring peaks, which slipped to a low-energy region, and the intensity of these peaks decreases with the increasing clay content. Accordingly, it can be said that polymer-organoclay interactions deformed the helixical structure of PEO chains. 

The band at 2855 cm^−1^ associated with symmetrical CH_2_ vibrations and it is understood that the above-mentioned peak has a tendency not to change in terms of both expansion and intensity increase with increasing organo-clay content. Accordingly, the diffusion of polymer chains to interlayer regions at lower clay ratios causes contraction from the spherical form to the oblate form, and this situation increases the freedom of movement of the tails of CTAB. This interaction weakens with the increase of the organo-clay content. 

**Figure 9:**
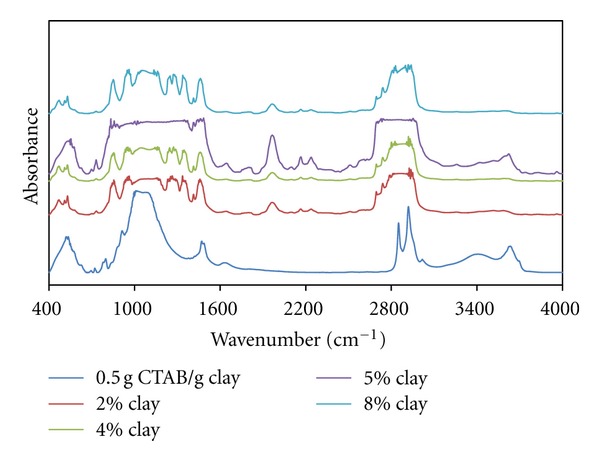
FTIR spectra of produced PEO/clay nanocomposites at various clay compositions (0.5 g CTAB/g clay ratio).

As can be seen in Figure 9, with the increasing clay content, the band at 2400 cm^−1^ associated with CH_2_ vibration and the vibration band expands, and this situation leads to the supposition that the intercalation of polymer chains leads to changes in micellar structure. As for the band at 910 cm^−1^, belonging to characteristic Si-O stretch vibration, it expands and splits appear which indicate the intense interaction between micellar structures and these groups. 

**Figure 10:**
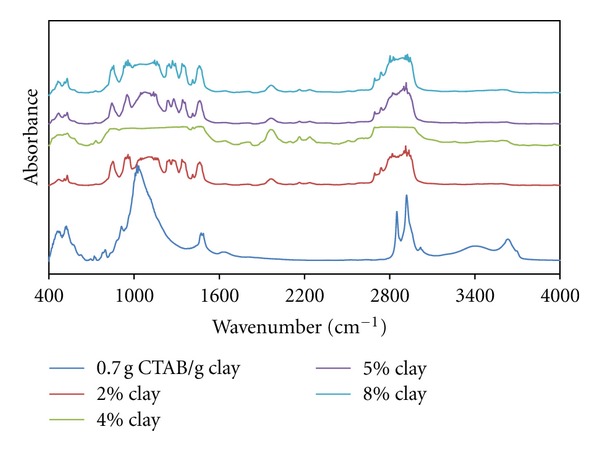
FTIR spectra of produced PEO/clay nanocomposites at various clay compositions (0.7 g CTAB/g clay ratio).

**Figure 11:**
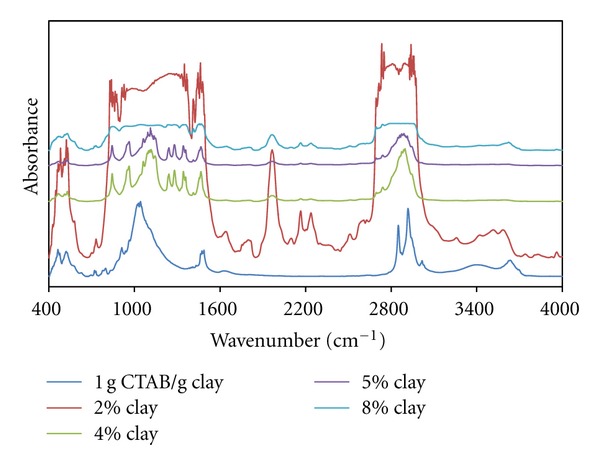
FTIR spectra of produced PEO/clay nanocomposites at various clay compositions (1.0 g CTAB/g clay ratio).

**Figure 12:**
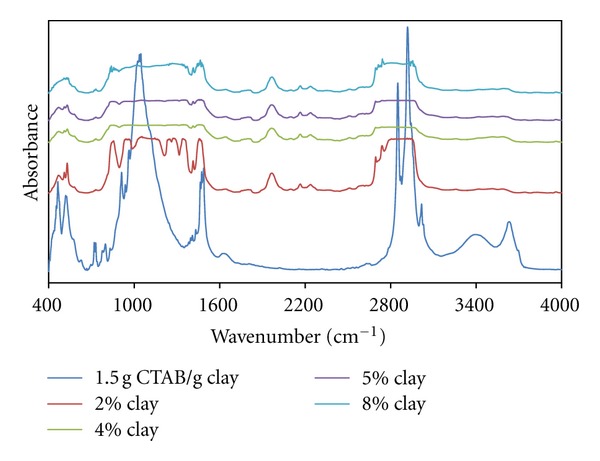
FTIR spectra of produced PEO/clay nanocomposites at various clay compositions (1.5 g CTAB/g clay ratio).

All figures (Figures 10, 11, and 12) show that the band associated with CH_2_ vibration from the tail parts of CTAB chains changes significantly with the increasing organoclay content and the characteristic bands associated with Si-O vibrations significantly widen. This reflects the intense interactions between micelles and the charged groups in interlayer regions, which also indicates the fact that the *a*_0_/*b*_0_ ratio of the micelles in oblate form increases. 

In addition, the spectrum for the sample with organo-clay of 2% content from the composites prepared with the sample of 1.0 g CTAB/g clay content reflects that these interactions have a high intensity. The FT-IR spectra of the sample with 1.5 g CTAB/g clay demonstrates that micellar adsorption leads to a high aggregation number. 

### 3.4. Mechanical Analysis

#### 3.4.1. The Tensile Strength

**Figure 13:**
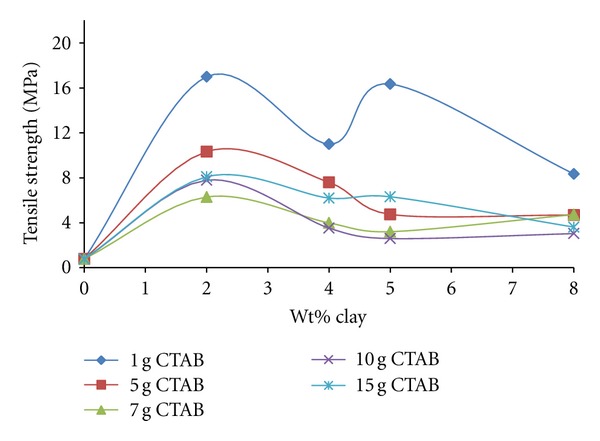
The change of tensile strength of produced PEO/clay nanocomposites at various CTAB/clay ratios with organo-clay contents.

The tensile strengths of PEO/organo-clay composites prepared with organo-clay in different CTAB-clay ratios increase when the content of clay increases to 2%. It then decreases and its highest values are 0.1 g CTAB/g clay ratio for all clay contents studied (see Figure 13). 

The same figure also suggests that the tensile strengths of the samples in ratios of 0.7 g CTAB/g clay and 1.0 g/g clay exhibit similar tendencies. The tensile strength for the sample in the ratio of 1.5 g CTAB/g clay is higher compared to the other two samples. 

It is realised that the polymeric peak intensity of the sample prepared with organo-clay with a ratio of 0.1 g CTAB/g clay decreases, and this situation indicates partial tactoidal structures according to the XRD diffract grams. Consequently, it can be argued that the interaction degree of exfoliated and tactoidal dispersion of organo-clay particles is very high. Therefore, the decrease in the tensile strength of the sample in the clay content of 4% can be related to the decrease in the exfoliation degree. 

The increase in the tensile strength may be connected with tactoidal dispersion of organo-clay particles into the polymer matrix. It can be claimed that the exfoliated clay layers with a high charge density create a disadvantage in terms of electrostatic repulsions. It is possible that the increased clay content makes favourable the ion-dipole interactions between the PEO chains and dispersed layers, although this increases the effectiveness of electrostatic repulsions. 

#### 3.4.2. Yield Strength

**Figure 14:**
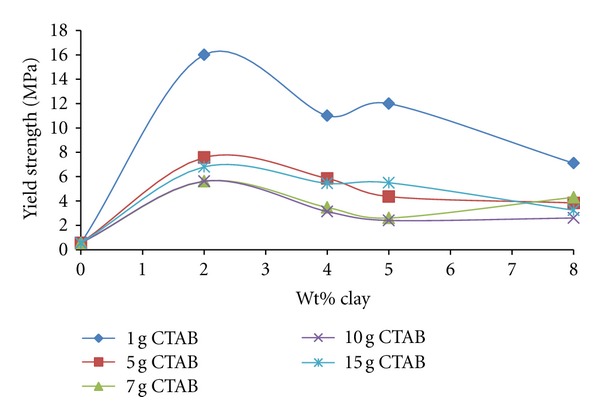
The change of yield strength of produced PEO/clay nanocomposites at various CTAB/clay ratios with organo-clay contents.

As can be seen in Figure 14, the yield strength of PEO/organoclay samples prepared by the use of organo-clays with different CTAB-clay ratios increases by 2% as the ratio of clay increases and then begins to decrease. As expected, the high dependency of yield strength values on clay content, in the case of 0.1 g CTAB/g clay negative charged exfoliated clay layers can deform the PEO chains. 

The exfoliated clay layers in which positive charged micellar groups at higher CTAB/clay ratios are adsorbed in a way that can cause disorders in the matrix and thus both tensile strength and yield strength decrease with the increased CTAB/clay ratio as well as the clay content. 

As could be expected, the increase in clay content negatively influences both the emergence of electrostatic interactions and also the strength values, depending on the change of orientation of polymer chains. 

#### 3.4.3. Strain Percentage

**Figure 15:**
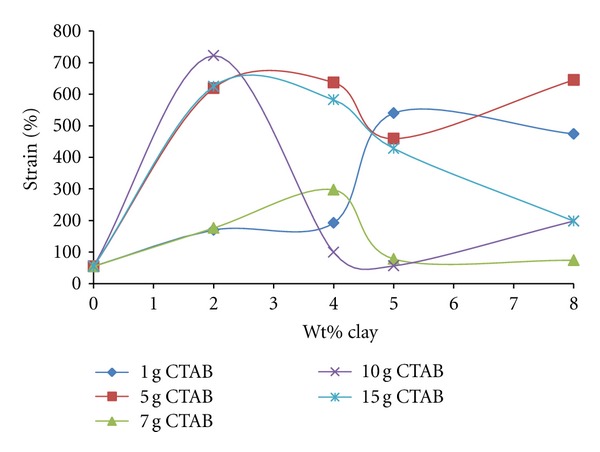
The change of values of strain percent of produced PEO/clay nanocomposites at various CTAB/clay ratios with organo-clay contents.

Figure 15 depicts that the strain percent values of PEO/organo-clay samples prepared using organo-clays with different ratios of CTAB-clay show very irregular changes in terms of both increasing clay content and increasing CTAB/clay ratios. As can be seen from XRD diffract grams, exfoliated dispersions at almost all ratios of CTAB/clay are predominant. These irregular changes can be attributed to a higher micelle aggregation number and its geometry in the case of higher CTAB/clay ratios.

The interactions between the organo-clay layers, in which the micelles are in the oblate form, with a potentially high aggregation number and high positive charge, the PEO chains, force the chains from a linear form to zigzag form. Therefore, the strain characteristics change depending on the mutual interactions of polymer chains of the sphere or ellipsoidal structure surrounding dispersed platelets. 

## 4. Conclusions

- Polyethylene oxide (PEO)/organo-clay nano composites were prepared using the solution intercalation method.
- The difference between the zeta potential values and the change in the interlayer distance of organo-clay was correlated using an empirical equation which is Δ*ξ* = 15.7Δ*d* + 68.6.
- From XRD analyses, it could be said that the increase in the exfoliation degree with the increase in the clay content makes diffusion of PEO chains into interlayer regions easier. In addition, the increased PEO diffusion forces the spherical micelles into lateral bilayer adsorption, which leads to more exfoliation because of the higher clay content. This may increase the occurring probability of both sphere-ellipsoid transition and tactoidal dispersion.
- The analysis of FTIR spectra show that the increase in intensity of Si-O stretch vibrations of organo-clay particles can be correlated with the partial decrease of micelle-Si-O interaction, because of the increase in the aggregation number of micelles formed at the increasing CTAB content and their geometry change. This can also be related to the transitions of spherical ellipsoidal (oblate) micelles, depending on the increase of the CTAB-clay ratio.
- The change in the mechanical properties of (PEO)/organo-clay nano composites, such as the tensile strength, the yield strength, and strain percent with CTAB/clay ratio and clay content of composites was investigated as a function of the zeta potential values.

## Figures and Tables

**Table 1:** Chemical composition of the MMT.

Components	Percentage
SiO_2_	59.3200
Al_2_O_3_	17.1900
Fe_2_O_3_	5.9490
MgO	3.6320
CaO	2.2110
Na_2_O	1.6670
K_2_O	0 .9732
TiO_3_	0.7436
SO_3_	0.5068
Other	7.8074
